# Mass Cytometry for Detection of Silver at the Bacterial Single Cell Level

**DOI:** 10.3389/fmicb.2017.01326

**Published:** 2017-07-17

**Authors:** Yuting Guo, Sabine Baumgart, Hans-Joachim Stärk, Hauke Harms, Susann Müller

**Affiliations:** ^1^Department of Environmental Microbiology, Helmholtz Centre for Environmental Research Leipzig, Germany; ^2^Department of Immune Monitoring, German Rheumatism Research Centre, An-Institute of the Leibniz Association Berlin, Germany; ^3^Department of Analytical Chemistry, Helmholtz Centre for Environmental Research Leipzig, Germany

**Keywords:** mass cytometry, metal-based cell marker, silver quantification in single cells, silver distribution, bacterial heterogeneity, silver nanoparticles

## Abstract

**Background:** Mass cytometry (Cytometry by Time of Flight, CyTOF) allows single-cell characterization on the basis of specific metal-based cell markers. In addition, other metals in the mass range such as silver can be detected per cell. Bacteria are known to be sensible to silver and a protocol was developed to measure both the number of affected cells per population and the quantities of silver per cell.

**Methods:** For mass cytometry ruthenium red was used as a marker for all cells of a population while parallel application of cisplatin discriminated live from dead cells. Silver quantities per cell and frequencies of silver containing cells in a population were measured by mass cytometry. In addition, live/dead subpopulations were analyzed by flow cytometry and distinguished by cell sorting based on ruthenium red and propidium iodide double staining. Verification of the cells’ silver load was performed on the bulk level by using ICP-MS in combination with cell sorting. The protocol was developed by conveying both, fast and non-growing *Pseudomonas putida* cells as test organisms.

**Results:** A workflow for labeling bacteria in order to be analyzed by mass cytometry was developed. Three different parameters were tested: ruthenium red provided counts for all bacterial cells in a population while consecutively applied cisplatin marked the frequency of dead cells. Apparent population heterogeneity was detected by different frequencies of silver containing cells. Silver quantities per cell were also well measurable. Generally, AgNP-10 treatment caused higher frequencies of dead cells, higher frequencies of silver containing cells and higher per-cell silver quantities. Due to an assumed chemical equilibrium of free and bound silver ions live and dead cells were associated with silver in equal quantities and this preferably during exponential growth. With ICP-MS up to 1.5 fg silver per bacterial cell were detected.

**Conclusion:** An effective mass cytometry protocol was developed for the detection and quantification of silver in single bacterial cells of different physiological states. The silver quantities were generally heterogeneously distributed among cells in a population, the degree of which was dependent on micro-environmental conditions and on silver applied either in ion or nanoparticle-aggregated form.

## Introduction

Increasing use of antimicrobial commercial products amended with silver nanoparticles (AgNP) has caused concerns, and extensive study was directed on AgNP toxicity of causing generation of reactive oxygen species (Choi and Hu, 2008; Franci et al., 2015), cell growth inhibition (Ivask et al., 2014), or cell viability loss (Guo et al., 2017). The characterization of AgNP physicochemical behavior with the focus on their dissolution, aggregation, and transformation in biological environments became increasingly important (Fabrega et al., 2011). As a result of these efforts recent studies suggested that dissolved silver ions can be held responsible for the antimicrobial qualities of AgNP (Xiu et al., 2012). We confirmed these findings in an own study but revealed an additional particle effect supposedly caused by fast formation of huge AgNP-aggregates of about 500 nm in cell solutions and suggested their contribution to higher cell death ratios (Guo et al., 2017). The attachment of AgNP-aggregates to single bacterial cells was identified by TEM and SEM-EDX (Guo et al., 2017). Aside from TEM and SEM-EDX, other techniques have been used to visualize and identify metals in single human cells, e.g., AFM, SXFM (Cerchiaro et al., 2013). However, these microscopy techniques have limitations including random sample detection, and lack of quantitative information regarding cellular interactions with nanoparticles. Flow cytometry has been reported to characterize single cells in microbial populations with high-throughput by means of light scatter and fluorescent dyes (Müller and Nebe-von-Caron, 2010). Cellular AgNP uptake was conveyed by this method via changes of side scatter characteristics but only for eukaryotic cells (Lankoff et al., 2012; Zucker et al., 2013; Zhao and Ibuki, 2015). For bacterial cells side scatter did not reveal any AgNP uptake (for sizes of 10 and 30 nm; Guo et al., 2017). Instead, ICP-MS has been operated widely to quantify the contents of silver ions in bacterial cells (Swathy et al., 2014; Wakshlak et al., 2015). It is a destructive technique where entire cell suspensions are digested and injected which prevents differentiation of silver-affected cells from unaffected ones. The ICP-MS obtained average value from a highly heterogeneous cell population disregards cell subsets and diverse phenotypes that may be relevant to reveal causality of cell reaction and toxicity in response to silver.

A growing interest in single-cell analysis can be recognized and numerous analytical methods have been developed or improved. One of those technologies is mass cytometry, which couples mass spectrometry with single-cell analysis and was introduced as Cytometry by Time of Flight (CyTOF) (Bendall et al., 2011; Chang et al., 2017). CyTOF offers numerous potential advantages over fluorescence-based flow cytometry, e.g., overcoming the challenge of spectral overlap intrinsic to fluorescent dyes by using rare-earth-metal stable isotopes with little signal overlap. In addition, more than 35 simultaneously measured cellular parameters compared to 10 markers in fluorescence-based flow cytometry can be measured (Bjornson et al., 2013; Chattopadhyay et al., 2014). To date this promising technique is mainly used for human cells as an effective tool for drug development or improvement of therapeutic programs ranging from infectious disease to cancer (Bendall et al., 2011; Gavasso et al., 2016; Robinson and Mao, 2016; Baca et al., 2017; Baumgart et al., 2017). Also other inorganic nanoparticles have been lables (Vancaeyzeele et al., 2007; Lin et al., 2014; Tong et al., 2016; Schulz et al., 2017) but were in the focus of biodistribution experiments (Yang et al., 2017). Limited studies have been reported on microbial cells (Leipold et al., 2011; Miyashita et al., 2014), where, e.g., a combination of a metal-based membrane stain and lectins, conjugated to lanthanide-chelating polymers, was used to differentiate *Escherichia coli* cells based on their cell surface polysaccharides.

In this study, we tested the mass cytometry technology for discrimination of live/dead cell states and simultaneous quantification of silver in single bacterial cells. An earlier study (Guo et al., 2017) revealed random attachment of huge up to 500-nm-AgNP-aggregates to a limited number of cells in a population after few minutes treatment of cells with 10- and 30-nm AgNP at environmental relevant concentrations. A relation between viability states and increased quantities of silver ions in cells by those AgNP-aggregates was suggested. Because flow cytometry does not allow direct detection of these two events simultaneously, a mass cytometry workflow was developed for the purpose. Such data may be especially useful to link cell states and features with cell fate and thus to contribute to the development of models that implement immanent characteristics of an individual cell and its individual capacity to notice random, selective, and perhaps lethal influences from the environment.

## Materials and Methods

### Materials

Silver nitrate (AgNO_3_) (99.9%) and ruthenium red (RR) was purchased from Sigma–Aldrich (United States). AgNPs were provided by nanoComposix (United States) as aqueous suspensions [citrate coated, mass concentration (Ag) 0.02 mg/mL] of the size 10 nm (9.4 ± 1.7 nm, AgNP-10). *Cis*-Platinum (II) diamine dichloride (cisPt) was purchased from Enzo Life Sciences GmbH (Lörrach, Germany). Nitric acid (HNO_3_) was purchased from Merck (Germany). M12 medium and PBS compositions were shown in Supplementary Table S1. For washing purposes 18.2 MΩ⋅cm water (MilliQ, Germany) was used.

### Bacterial Cultivation

*Pseudomonas putida* KT2440 was obtained from the German Collection of Microorganisms and Cell Cultures (DSMZ, Germany). Bacterial standard-growth was performed in M12 medium on a rotary shaker at 30°C and 170 rpm. The growth was monitored by optical density at λ = 600 nm (Spectra max Plus 384 photometer, Molecular Devices, Sunnyvale, CA, United States).

### Bacterial Cultivation under Silver Treatment

An overnight pre-culture of *P. putida* KT2440 was incubated in M12 medium with an initial OD_600_ of 0.09 and grown for 72 h (30°C, 170 rpm). Either AgNP-10 (1.29 mg/L) or AgNO_3_ (0.19 mg/L) were implemented in the cultivations and chosen concentrations referred to the determined EC_50_ values from an earlier publication (Guo et al., 2017). Cultivations without silver treatment served as silver-ion negative control while application of AgNO_3_ served as silver-ion positive control. Cells were harvested at 0, 12, 48, and 72 h and treated separately according to the mass cytometry staining protocol (see below).

### Determination of Cell Number

To analyze bacteria on the single cell level at the mass cytometer, a concentration of 5.0 × 10^5^ cells/mL was required for each injection. Therefore, a fast and accurate cell counting method was required and for this a range of linear relationship between cell counts and OD_600_ was exploited. Cell counts were determined by a flow cytometer (Becton, Dickinson and Company, Franklin Lakes, NJ, United States) together with a calibrated suspension of microsphere standard (6.0 μm diameter microspheres at a concentration of 10^8^ beads/mL in Milli-Q water containing 2 mM sodium azide, L34856, Thermo Fisher Scientific, Germany) for accurate cell count measurements. OD_600_ was analyzed by a spectrophotometer. All measurements were done in replicates and shown in Supplementary Figure S1.

### All Cell Indicator for Mass Cytometry

To optimize RR staining for *P. putida* KT2440 populations, a stock solution of 1.3 mM of RR in PBS was prepared and stored at 4°C. Before use the solution was ultra-sonicated (Ultrasonic bath, Merck Eurolab, Darmstadt, Germany) for 10 min. 10^8^ cells were treated with different concentrations of RR (0 to 0.33 μM) for 30 and 60 min. The final staining protocol requires 0.13 μM RR/10^8^ cells/200 μL PBS and a staining time of 30 min at room temperature (RT). Details of staining optimization were shown in Supplementary Figure S2A.

### Dead Cell Indicator for Mass Cytometry

To optimize cisPt staining for *P. putida* KT2440 populations, a stock solution of 25 mM of cisPt in dimethyl sulfoxide (Sigma–Aldrich, United States) was prepared and stored at -20°C. 10^8^ cells were stained with different concentrations of cisPt (0 to 20 μM) and treated for 1, 5, 10, 30 min. The final staining protocol requires 5 μM cisPt/10^8^ cells/1 mL PBS and a staining time of 10 min at RT. Details of staining optimization and calibration curves generated on the basis of RR and cisPt measurements were shown in Supplementary Figures S2B,C.

### Dead Cell Indicator for Flow Cytometry

The fluorescent dye propidium iodide (PI) was used as dead cell indicator for *P. putida* KT2440. Staining optimization and calibration were published (Guo et al., 2017). The final protocol requires 2 μM PI/10^8^ cells/1 mL PBS and a staining time of 2 min. For quantification of PI stained dead cells (PI+dead) of RR labeled *P. putida* KT2440, cells were double stained with RR (0.13 μM, 30 min), followed by PI (2 μM, 2 min) and analyzed by flow cytometry at log scale.

### Workflow for Analysis of Bacterial Cells at the Mass Cytometer

Harvested cells were diluted to 10^8^ cells/1 mL PBS and centrifuged at 3200 × *g* for 10 min. The pelleted cells were re-suspended in cisPt solution (5 μM, 10 min, RT). Subsequently, the cisPt stained dead cells (cisPt+dead) were washed (3200 × *g*, 10 min) twice with 3 and 1 mL PBS, respectively, to remove unbound cisPt. Finally, cells were stained with RR (0.13 μM, 30 min, RT), washed twice (3200 × *g*, 10 min) with 1 mL water to remove salts and unbound RR. For mass cytometry measurement the cell concentration was adjusted to 5.0 × 10^5^ cells/mL in Milli-Q water. Four element calibration beads (Fluidigm, United States) were added 1:10 v/v before acquisition for later normalization (Finck et al., 2013).

### Mass Cytometry

The CyTOF instrument (Fluidigm Corp, South Francisco, CA, United States) was tuned, calibrated, and cleaned on the daily base according to the manufacturer’s advice. Aqueous bacterial cell suspensions were acquired on a CyTOF instrument version 1 upgraded to the control software of v6.0.626 with a flow rate of 45 μL/min. Argon gas 5.0 was used to generate the plasma and for nebulizing the cell suspension. About 1.5 × 10^5^ cells per sample were subsequently loaded onto a 450 μL sample loop and measured in a dual instrument mode with noise reduction turned on, a cell length range from 1 to 75 and with ‘on the fly’ processing. The initial raw data were processed into standard FCS file format, randomized and normalized by Software Helios version 6.5.358 (Fluidigm Corp, South San Francisco, CA, United States). Data analysis was performed with FlowJo (version 10) (TreeStar, Ashland, OR, United States). Monitoring was done by analyzing natural abundance isotopes of ruthenium (^102^Ru and ^104^Ru), platinum (^195^Pt) and silver (^107^Ag and ^109^Ag, 51.8 and 48.2%, respectively). Silver was measured in ionic form and resulting data were related to the ^107^Ag isotope.

### Flow Cytometry and Cell Sorting

Cytometric measurements were performed with a BD Influx v7 Sorter USB, (Becton, Dickinson and Company, Franklin Lakes, NJ, United States) equipped with a blue 488-nm Sapphire OPS laser (400 mW, Coherent, Santa Clara, CA, United States). The 488-nm laser light was used for the analysis of the forward scatter (FSC, 488/10), the side scatter (SSC, 488/10, trigger signal), and the PI induced red fluorescence (616/23). The fluidic system was run at 33 psi using a 70-μm nozzle. The sheath fluid consisted of 0.5 × FACSFlow buffer (BD). For the optical calibration of the cytometer in the linear range, 1-μm blue fluorescent FluoSpheres (Molecular Probes, F-8815, Eugene, OR, United States) and 2-μm yellow-green fluorescent FluoSpheres (Thermo Fisher Scientific, F8827, Waltham, MA, United States) were used. For calibration in the log range, 0.5-μm UV Fluoresbrite Microspheres (Polysciences, 18339, Warrington, PA, United States) were applied. Sorting of PI unstained live (PI-live) and PI stained dead (PI+dead) cells into plastic tubes was performed at an event rate of 5.000/sec which corresponded to a sort rate of 700–1.500 cells/sec. The sort mode was 1.0 Drop Pure. To obtain sufficient cell amounts for the following mass cytometry and ICP-MS measurements, up to 3.0 × 10^6^ cells were sorted for either PI-live or PI+dead cells. After sorting cells were pre-processed before CyTOF or ICP-MS analysis: sorted cells were transferred from plastic tubes into glass tubes and centrifuged at 3200 × *g* for 10 min, supernatant was removed and the cell pellets were stored at -20°C. Before mass cytometry measurements, cell pellets were washed once with 1 mL Milli-Q water by re-suspension in 1 mL Milli-Q water for injection.

### ICP-MS Analysis

For bulk ICP-MS analysis, the sorted PI-live and PI+dead cell pellets were digested by addition of 50 μL HNO_3_ (60%, ultrapure) for a period of 15 min. Subsequently the solution was suspended in Milli-Q water dispensed with rhodium (^103^Rh) as internal standard [c (Rh) = 0.75 ng/mL] to a 1 mL solution. The solution was measured with an Inductively Coupled Plasma Mass Spectrometer (ICP-MS, Element XR, Thermo, Germany) equipped with a micro-concentric nebulizer (Micromist-100, Glass Expansion, Australia) consuming 135 μL sample per minute. Silver was measured and quantified in ionic form related to the ^109^Ag isotope.

## Results and Discussion

### Bacterial Labeling for Mass Cytometry Analysis

Cytometry by Time of Flight technology substitutes isotopes of transition elements and lanthanides for fluorescent dyes. The cells are directed into a narrow flow to be screened one by one and vaporized. Elements of the cells are atomized, ionized, and subsequently analyzed by a time-of-flight mass spectrometer. The measured signal of natural abundance isotopes of the rare-earth-metals ruthenium and platinum per cell from applied RR and cisPt provided the number of labeled cells and the isotopes’ quantity per cell. RR is a cationic reagent and has been widely used to locate acidic polysaccharide-like material (Fletcher and Floodgate, 1973; Fassel and Edmiston, 1999; Waller et al., 2004) and to visualize numerous ultra-structural details in and outside of cells (Lingens et al., 1985; Chatterjee et al., 2010; Perfumo et al., 2014). Because RR is easy to operate by addressing all cells in a population directly without pre-treatment (instead of, e.g., fixation, necessary for flow cytometric measurements; Günther et al., 2008) and its atomic mass is within the detectable mass range of mass cytometers, RR was used as an all cell indicator for the CyTOF workflow. However, the metal was not used in this context for bacteria before. The second dye used in this study, cisPt, is a readily available platinum based chemotherapeutic agent and reacts with protein nucleophiles, with which it can form covalent platinum–sulfur or sulfhydryl bonds (Fienberg et al., 2012). cispt enters cells with compromised cell membranes, where it non-specifically labels total cellular protein. The reagent can thus be used to discriminate live from dead cells which is well practiced for human cells (Bjornson et al., 2013; Nair et al., 2015). However, applications on bacteria haven’t been tested yet to our knowledge.

**FIGURE 1:**
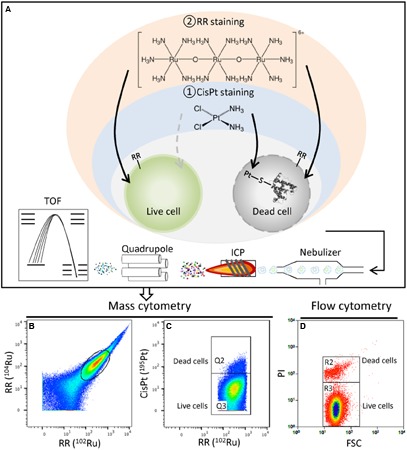
**(A)** CyTOF workflow for bacteria. Cells were first stained by cisPt for live/dead discrimination, followed by RR staining as an overall bacterial cell indicator. After washing, cells were subjected to the mass cytometer as a stream of single cells. In the plasma region elements of the cells are atomized and ionized. Ions of high-mass elements (i.e., ruthenium, platinum, and silver) enter selectively the TOF chamber and are separated according to their mass before detection. **(B)** CyTOF analysis of standard-grown and harvested *P. putida* KT2440 (48 h). CyTOF plots marked all events based on ^102^Ru/^104^Ru signal. **(C)** Dead cisPt+ (Q2) cells are distinguished from live cisPt– (Q3) cells. **(D)** Dead (R2) and live (R3) cells are distinguished by PI staining and flow cytometry.

In our study, the Gram-negative bacterium *P. putida* KT2440 was used as a test organism to establish a protocol for RR and cisPt staining in order to detect all cells in a population and to differentiate live from dead cells. The RR stained bacteria were well detectable by the CyTOF and entirely identified within a cell gate plotted via the two most natural abundance ruthenium isotopes ^102^Ru/^104^Ru. Protein binding cisPt was used as second cell marker for dead cells and identified by the ^195^Pt isotope. For this combined marker group optimized staining conditions were tested with regard to dye concentrations and times of incubation and the trade-off between highest intensity and shortest staining time was chosen for the final workflow (**Figure 1A** and Supplementary Figure S2B). To verify the reliability of the cisPt marker different ratios of live and dead cells (70% ethanol treated, 20 min, RT) were prepared and measured at CyTOF. In parallel the same ratios were analyzed by using the fluorescent marker PI (binding on nucleic acids) for dead cells and flow cytometry. While flow cytometry mirrored the precise ratios, CyTOF gave lower dead cell counts (Supplementary Figure S2C). Up to now the dye was only applied to cell-wall-free cells such as human cells effectively (Majonis et al., 2011; Fienberg et al., 2012). Bacterial cells, however, have rigid cell walls, subsistent also in dead cells, forming a barrier which must be overcome by cisPt. It can be assumed that the limitation in the quantitative detection of dead cells in a population may be caused by this boundary. Nevertheless, despite the determination of dead cell numbers by cisPt in a population of *P. putida* KT2440 was lower in comparison to PI staining (Supplementary Figure S2C: *k_cisPt_* = 0.45, *k_PI_* = 0.98), the stable isotopes’ calibration of RR and cisPt proved to be useful cell markers. Standard-grown *P. putida* KT2440 cells (48 h) were represented in typical 2D-plots for RR staining (**Figure 1B**). The number of dead cells was 2.7% (Q2) measured by cisPt and mass cytometry (**Figure 1C**), and 4.1% (R2) measured by PI and flow cytometry (**Figure 1D**).

### Detection of Silver of Single Cells of *P. putida*

*Pseudomonas putida* KT2440 is known to react on various silver concentrations by changes in growth rates and live/dead cell ratios. The toxicity of silver was recently assumed to be caused by the ions only (Xiu et al., 2012; Giao et al., 2017; Juganson et al., 2017) but we detected an additional particle-related effect (Guo et al., 2017). We assumed that the typical fast aggregation of AgNPs to huge complexes in a nature-like environment within 30 min (Guo et al., 2017) might contribute to a further increase of dead cell counts due to their random attachment to bacterial cell surfaces and their thereby steady release of additional silver ions into those cells.

**FIGURE 2:**
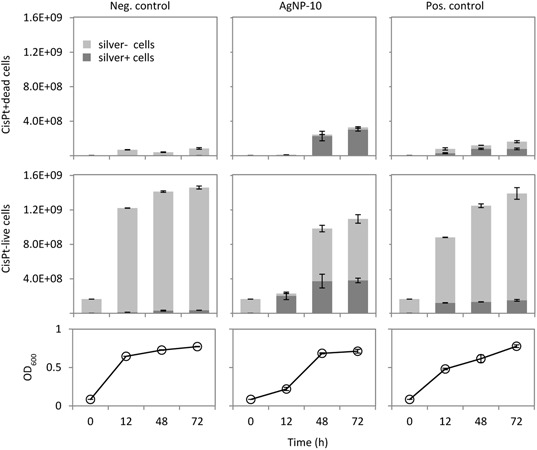
CyTOF measurements on cisPt-live/cisPt+dead cell discrimination and the number of silver containing cells in a population. *P. putida* KT2440 cells were cultivated in M12 medium with AgNP-10 (1.29 mg/L), with AgNO_3_ (0.19 mg/L) as positive control, and untreated as negative control. Growth curves were shown for 72 h by measuring OD_600_. Cells were harvested at 12, 48, 72 h and measured by CyTOF. Light gray bars showed cells without silver load, while dark gray bars showed silver containing cells. Standard errors were from replicate analysis.

Thus, mass cytometry was involved in this study to clarify if the treatment of bacteria with either silver ions or AgNP (1) generates different frequencies of cells that contain silver which may cause different live/dead cell ratios and (2) produces disparate quantities of silver per cell suggesting that populations exposed to AgNP might contain cells with a higher silver load. To answer these questions *P. putida* KT2440 cells were treated with the respective EC_50_ concentrations of 1.29 mg/L for AgNP-10 and 0.19 mg/L for AgNO_3_ as the positive control. As determined before, the released ion concentrations were nearly similar because cell-free AgNP-10 dissolution kinetic curves inferred 0.08 mg/L silver ions released from AgNP-10 (1.29 mg/L) in comparison to 0.12 mg/L silver ions from AgNO_3_ (0.19 mg/L) over a time period of 72 h (Guo et al., 2017). Standard-grown *P. putida* KT2440 without silver treatment served as the negative control. The growth curves (**Figure 2**, bottom) showed a delayed lag-phase for the AgNP-10 treated bacteria and a slower growth rate (μ = 0.14/h) for the AgNO_3_ positive control in comparison to the negative control (μ = 0.18/h). CyTOF technology was used to mark all bacterial cells of those populations and to distinguish live from dead cells. The additional measured silver signal was used to determine the frequency of silver containing cells as well as the quantities of silver per cell via the intensity of the mass cytometric signal for silver (IAg).

After 72 h and in comparison to the negative control AgNP-10 treatment caused an increase of cisPt+dead cells by a factor of about 4 while the AgNO_3_ positive control showed a factor of 2 (**Figure 2**), indicating that the particles caused higher frequencies of dead cells. Further, the frequency of cells that contain silver (**Figure 2**, dark gray bars) was also higher for the AgNP-10 treatment in comparison to the positive control while cells of the untreated negative control were nearly not loaded with silver (**Figure 2**). After 72 h AgNP-10 treatment 92% of cisPt+dead cells and 35% of cisPt-live cells were loaded with silver. Instead, the AgNO_3_ positive control revealed 48% silver containing cells of the cisPt+dead and only 11% of the cisPt-live cells (**Figure 2**). The data show that AgNP-10 treatment caused the highest frequencies of dead cells in comparison to both controls after 72 h, and that almost all of the dead cells were loaded with silver. At the beginning of the exponential phase (12 h) where cells thrived at merely low numbers, live cells were still predominant but silver was loaded to any cell independent of its viability state. At longer exposure time the high frequency of silver loaded dead cells was almost consistent between 48 and 72 h. A similar tendency was observed within the larger fraction of surviving silver containing cells, indicating that the absorbed silver (ions) do not cause immediate cell death.

We also determined silver quantities per cell by measuring their IAg values which were different between AgNO_3_ positive control and AgNP-10 treated cells (**Table 1**). Overall, AgNP-10 treatment led to much higher silver quantities per cell in comparison to the positive control. Our data also suggest that the AgNP-aggregates, formed asap from AgNP-10, were not present at all or at least in their entirety due to the stringent washing procedures before CyTOF measurement otherwise we would expect an elevated silver signal in the 1D-plot of the silver channel (Supplementary Figure S3). But the silver ions released earlier by AgNP-aggregates and then bound by the cells were obviously responsible for the higher silver contents in AgNP-10 treated cells. In addition, for AgNP-10 treated cells we found nearly identical IAg values for both cisPt-live and cisPt+dead cells when cells grew exponentially (12 h: 18.3 ± 0.6 vs. 17.1 ± 1.1, **Table 1**) which was different for early and late stationary cells where the IAg values were higher for the cisPt+dead cells (e.g., for 72 h: 1.6 ± 0.1 vs. 10.3 ± 0.6). For AgNO_3_ treated cells the IAg values per cell were generally much lower and increased finally to only 3.0 ± 0.3 after 72 h for cisPt+dead cells. Thus, silver quantities per cell were higher under AgNP-10 treatment with the highest IAg values determined for cells from exponential phase independent if they were dead or alive.

**Table 1:** The silver quantities per cell via the intensity of the mass cytometric signal for silver (IAg).

Time (h)	Treatment	IAg	
		cispt-live cells	cispt+dead cells
12	Pos. control	0.5 ± 0.0	0.9 ± 0.1
	AgNP-10	18.3 ± 0.6	17.1 ± 1.1
48	Pos. control	0.3 ± 0.0	3.4 ± 0.3
	AgNP-10	2.1 ± 0.0	11.0 ± 0.5
72	Pos. control	0.3 ± 0.0	3.0 ± 0.3
	AgNP-10	1.6 ± 0.1	10.3 ± 0.6


*P. putida KT2440 cells were cultivated in M12 medium with AgNP-10 (1.29 mg/L) and with AgNO_3_ (0.19 mg/L) as positive control for 72 h. Cells were harvested at 12, 48, 72 h and measured by CyTOF. Median values for intensities of ^107^Ag (IAg) in cisPt-live and cisPt+dead cells were shown. Examples of CyTOF plots were in Supplementary Figure S3*.

The data of both the calculated frequencies in cell numbers and the silver quantities per cell follow the same trend. AgNP-10 treatment cause higher frequencies of dead cells, higher frequencies of silver affected cells and higher per-cell silver quantities whereby live and dead cells load silver in equal quantities and this preferably during exponential growth.

Independent from this, the population showed heterogeneous cell states with respect to silver quantities which might simply be caused by chemical equilibria of available free silver ions. Although we were not able to distinguish a silver signal related to cell-attached AgNP-aggregates or to distinguish aggregate-dissolved silver ions from free silver ions with CyTOF technology we assume that by a fast process an equilibrium is established and leads to the silver ions’ equal distribution among cells. When the equilibrium changes in favor of the cells, because of increasing cell numbers during growth, these mostly new live cells were found free of silver. This observation was made also with AgNO_3_ treated cells. It can also be assumed that a continuous uptake of silver ions by the cells may influence the equilibrium between AgNP-aggregate bound and unbound silver ions in the medium and cause further dissolution of silver ions from the aggregates (Yue et al., 2017).

The equal silver quantities in both live and dead exponential grown cells were verified by sorting identical numbers (3 × 10^6^ cells each) of PI unstained/PI stained cells, from which an aliquot of cells of each sample were injected into the mass cytometer and 10^6^ cells of each sample was used for ICP-MS measurements. As before, the cells were treated by AgNP-10 at EC_50_ dosage of 1.29 mg/L. From CyTOF analysis we found analogous silver quantities per cell among PI-live and PI+dead cells with IAg = 21.6 and IAg = 17.9, respectively. The equal silver quantities per cell were by tendency confirmed by the ICP-MS results with 1.5 fg and 1.1 fg silver per PI-live and PI+dead cells, respectively. Overall, the equal silver quantity distribution among live and dead cells suggested that random AgNP-aggregate attachment is not only affecting and toxic to an appended cell but also to every other cell in its vicinity.

## Conclusion

The primary objective of this study was to establish a mass cytometry method for the analysis of bacteria exemplary taking the Gram-negative bacterium *P. putida* KT2440. Our data show that by using RR, mass cytometry is able to detect all bacteria in a population, and that, by combining RR with cisPt, the method differentiates live from dead bacterial cells. The panel was extended by including natural abundance silver isotopes in order to measure the silver level in a single bacterial cell.

In Guo et al. (2017), PI uptake was correlated with silver-cell toxicity. This proof, however, was indirect. Now, mass cytometry was not only able to confirm the data of the earlier study by a direct measurement of silver per cell but provided further findings. AgNP-10 treatment caused a two times higher number of dead cells in comparison to AgNO_3_ treatment after 72 h cultivation and three times higher numbers of cells with silver load. The per-cell silver quantities were also higher in AgNP-10 treated cells. Therefore, mass cytometry clearly supported the earlier discussion of a particle effect in addition to the toxicity of silver ions. The origin of this additional silver can be assumed to come from the AgNP-aggregates. In addition, the heterogenic distribution of silver ions was verified but seems to be independent on live/dead cell states but rather on existing chemical equilibria in the environmental vicinity. Thus, the action of the silver is clearly dependent on complex effects. Prediction of toxicant behavior to cells in natural environments will be even more complex due to the presence of further affecting parameters and can probably only be predicted when models implement equilibria of chemical components and their bioavailability and adjoin such data with the heterogeneous state or feature of single cells.

## Author Contributions

YG designed and conducted the experiments, evaluated the data and wrote the paper. SB analyzed samples for mass cytometry, helped to evaluate the data, and contributed to writing. H-JS analyzed samples for ICP-MS, helped to evaluate the data, and contributed to writing. HH contributed to writing. SM designed the experiments, evaluated the data and wrote the paper.

## Conflict of Interest Statement

The authors declare that the research was conducted in the absence of any commercial or financial relationships that could be construed as a potential conflict of interest.
