# Supplementary material for: Mass Cytometry for Detection of Silver at the Bacterial Single Cell Level
Source: Front Microbiol. 2017 Jul 17;8:1326. doi: 10.3389/fmicb.2017.01326 (PMC5511850; doi:10.3389/fmicb.2017.01326)
Supplement: Supplementary file 1 [file Data_Sheet_1.DOCX]

**Mass cytometry for detection of silver at the bacterial single cell level**

Yuting Guo^1^, Sabine Baumgart^2^, Hans-Joachim Stärk^3^, Hauke Harms^1^, Susann Müller^1^*

^1^Department of Environmental Microbiology, Helmholtz Centre for Environmental Research, Leipzig, Germany

^2^Department of Immune Monitoring, German Rheumatism Research Centre, Berlin, An-Institute of the Leibniz Association, Germany

^3^Department of Analytical Chemistry, Helmholtz Centre for Environmental Research, Leipzig, Germany

*Correspondence to: Susann Müller, E-mail: [susann.mueller@ufz.de](mailto:susann.mueller@ufz.de)

**Table of Contents**

[Table S1. Medium components for *P. putida* KT2440 cultivation 2](#_Toc481144384)

[Figure S1. Calibration curve between cell counts and OD_600_ 2](#_Toc481144385)

[Figure S2. Cell indicator for CyTOF: ruthenium red and cisplatin staining optimization 2](#_Toc481144386)

[Figure S3. CyTOF analysis plots 3](#_Toc481144387)

# Table S1. Medium components for *P. putida* KT2440 cultivation

|  |  | Component | g/L |
| --- | --- | --- | --- |
|  | Minerals | (NH_4_)_2_SO_4_ | 2.2 |
|  |  | MgSO_4_$\times$7H_2_O | 0.4 |
|  |  | KH_2_PO_4_ | 2 |
|  | Trace elements | Na-citrate | 0.015 |
|  |  | FeSO_4_$\times$7H_2_O | 0.01 |
|  | Carbon source | Glucose | 1 |

PBS composition: Na_2_HPO_4_ 0.85 g/L, NaH_2_PO_4_ 0.22 g/L, NaCl 8.5 g/L, pH 7.2.

# Figure S1. Calibration curve between cell counts and OD_600_

# Figure S2. Cell indicator for CyTOF: ruthenium red and cisplatin staining optimization

To optimize RR staining fixed *P. putida* KT2440 cells (in 2 % PFA 30 min, washed and re-suspended in 70 % ethanol) were used from a stationary phase sample. The cells were treated with a 4°C stock solution of 1.3 µM RR in PBS. 0 to 0.33 µM of RR was tested on 10^8^ cells / 200 µL PBS for both 30 and 60 min. The final staining protocol requires 0.13 µM RR / 10^8^ cells / 200 µL PBS and a staining time of 30 min.

To optimize cisPt staining a mixture of 50 % live and 50 % dead cells of a stationary phase *P. putida* KT2440 sample was used. The cells were treated with a -20°C stock solution of 25 mM cisPt. 0 to 20 µM of cisPt were tested on 10^8^ cells / 1 mL PBS and treated for 1 to 30 min. Following, the cells were treated with RR according to the optimized method described above. The final staining protocol requires 5 µM cisPt / 10^8^ cells / 1 mL PBS and a staining time of 10 min.


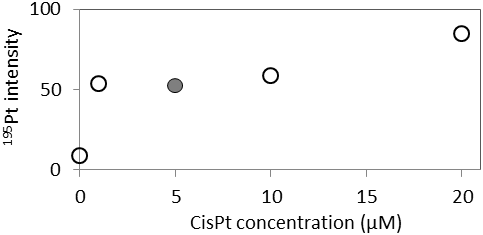

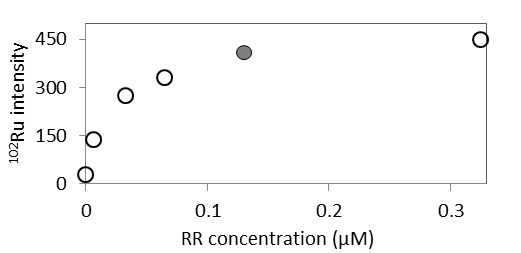


^102^Ru

^195^Pt


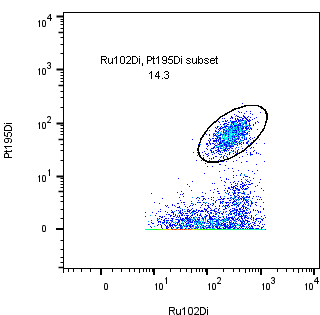

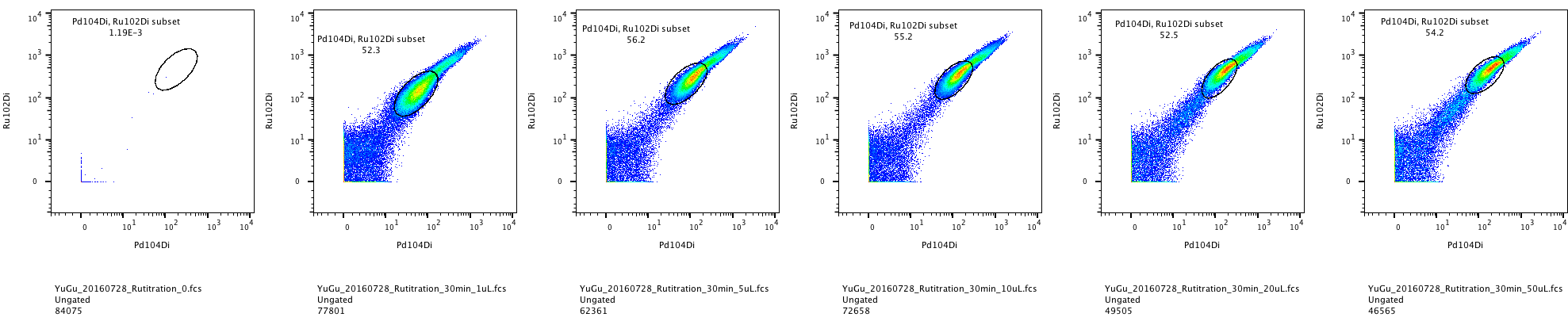


^104^Ru

^102^Ru

A

B

A calibration curve based on RR and cisPt measurements

Different ratios of live and 70 % ethanol treated dead cells (20 min, room temperature) were prepared, stained by cisPt+RR, and measured at CyTOF. In parallel the same ratios of cells were analyzed by using PI and flow cytometry. Standard errors were from replicate analysis.


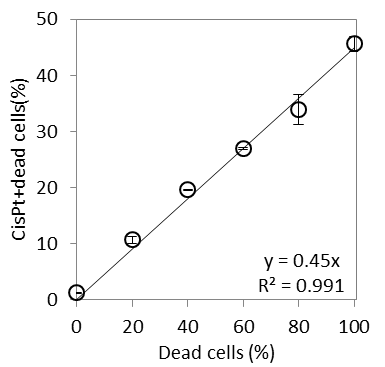

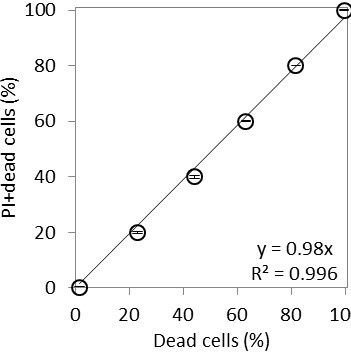


C

# Figure S3. CyTOF analysis plots

*P. putida* KT2440 cells were cultivated in M12 medium and treated with AgNP-10 (1.29 mg/L). Examples showed cells harvested at 12 h and 72 h, and analyzed by CyTOF. From left to right, first column: all cell events were marked based on ^102^Ru/^104^Ru intensity; second column: the live/dead cells were discriminated based on ^195^Pt intensity; third column: the counts of dead cells containing silver, forth column: the counts of live cells containing silver. The related intensity of silver (IAg, median) in cisPt+dead and cisPt-live cells was shown in Table 1.


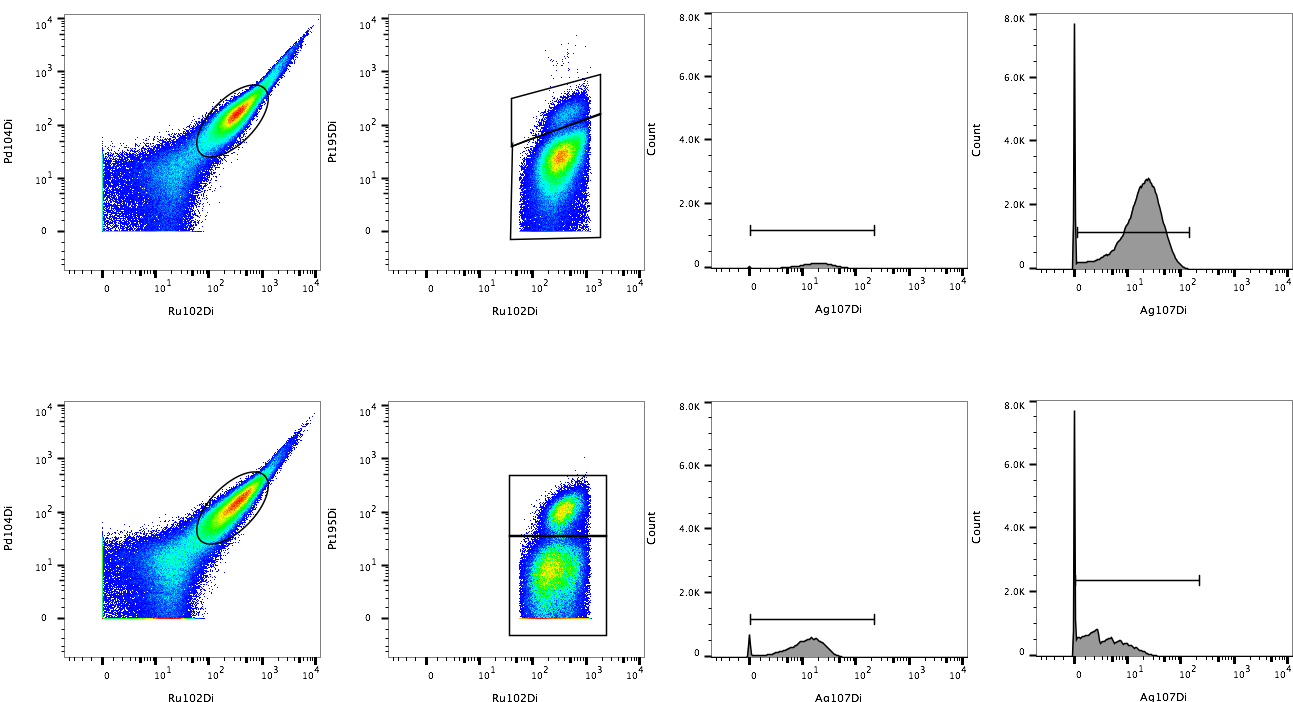


^102^Ru

^102^Ru

^102^Ru

^102^Ru

^107^Ag

^107^Ag

^107^Ag

Dead

Live

Dead

Live

^107^Ag subset:

Mean: 22.5

Median: 18.1

^107^Ag subset: Mean: 24.0

Median: 19.9

^107^Ag subset: Mean: 14.1

Median: 12.0

^107^Ag subset: Mean: 5.33

Median: 3.08

12 h

AgNP-10

72 h

AgNP-10

Counts

Counts

Counts

Counts

^104^Ru

^104^Ru

^195^Pt

^195^Pt

Dead

Live
